# The relationship of blue crab (*Callinectes sapidus*) size class and molt stage to disease acquisition and intensity of *Hematodinium perezi* infections

**DOI:** 10.1371/journal.pone.0192237

**Published:** 2018-02-23

**Authors:** Kristen A. Lycett, J. Sook Chung, Joseph S. Pitula

**Affiliations:** 1 Department of Natural Sciences, University of Maryland Eastern Shore, Princess Anne, Maryland, United States of America; 2 Department, The Institute of Marine & Environmental Technology, University of Maryland Center of Environmental Sciences, Baltimore, Maryland, United States of America; Zhejiang University College of Life Sciences, CHINA

## Abstract

In the blue crab, *Callinectes sapidus*, early studies suggested a relationship between smaller crabs, which molt more frequently, and higher rates of infection by the dinoflagellate parasite, *Hematodinium perezi*. In order to better explore the influence of size and molting on infections, blue crabs were collected from the Maryland coastal bays and screened for the presence of *H*. *perezi* in hemolymph samples using a quantitative PCR assay. Molt stage was determined by a radioimmunoassay which measured ecdysteroid concentrations in blue crab hemolymph. Differences were seen in infection prevalence between size classes, with the medium size class (crabs 61 to 90 mm carapace width) and juvenile crabs (≤ 30 mm carapace width) having the highest infection prevalence at 47.2% and 46.7%, respectively. All size classes were susceptible to infection, although fall months favored disease acquisition by juveniles, whereas mid-sized animals (31–90 mm carapace width) acquired infection predominantly in summer. Disease intensity was also most pronounced in the summer, with blue crabs > 61 mm being primary sources of proliferation. Molt status appeared to be influenced by infection, with infected crabs having significantly lower concentrations of ecdysteroids than uninfected crabs in the spring and the fall. We hypothesize that infection by *H*. *perezi* may increase molt intervals, with a delay in the spring molt cycle as an evolutionary adaptation functioning to coincide with increased host metabolism, providing optimal conditions for *H*. *perezi* propagation. Regardless of season, postmolt crabs harbored significantly higher proportions of moderate and heavy infections, suggesting that the process of ecdysis, and the postmolt recovery period, has a positive effect on parasite proliferation.

## Introduction

Organisms of the genus *Hematodinium* are the causative agent of a disease with a spectrum of manifestations, such as ‘Bitter Crab Disease’, ‘Pink Crab Disease’, and ‘Milky Crab Disease’ [1–3]. These parasitic dinoflagellates cause significant mortality in crustacean fisheries and aquaculture systems [4–6]. The mechanism for disease transmission is unknown, though a waterborne infective dinoflagellate stage is currently favored [7–10]. The peak of disease manifestation varies from host to host, and even between geographic locations within the same host species [1, 11–19]. Infection prevalence by season is suggested to be related to various environmental factors such as the salinity, temperature, pH, dissolved oxygen, and chlorophyll a levels in water [19–23]. Physiological conditions, principally the molt cycle of host crustaceans, have also been implicated in disease cycles [14, 15, 24].

In cold water host species, such as the Alaskan Tanner Crab (*Chionoecetes bairdi*) and the related Newfoundland snow crab (*C*. *opilio*), a relationship between molt status and infection by *Hematodinium* spp. is relatively clear. In these species, ‘new shell’ crabs are defined as those in Shell Condition 1 or 2, which have molted within a year of examination and possess shells that are lighter in color, lack epibionts, and are of minimum hardness [24, 25]. These new shell crabs are typically observed with much higher rates of infection compared to older shell crabs, and peak infection prevalence is typically observed in smaller size classes [11, 24, 25]. As over 98% of infected crabs are usually observed in Shell Condition 2 animals, it has been postulated that crabs acquire disease either during molting or in post-molt stages [24, 26].

By contrast the blue crab (*Callinectes sapidus*) molts at a higher rate, particularly in warmer summer months, and early studies suggested a tenuous link between molting and infection [27]. In a seminal study, juvenile crabs (< 30 mm carapace width [CW]) showed significantly higher *H*. *perezi* infection prevalence than larger size classes [14]. As smaller crabs molt more rapidly than larger size classes, the authors hypothesized that this observation could be explained by greater frequency of parasite exposure due to breaks in the carapace induced by molting. However, they also noted that infection intensity was not significantly different by size class, nor was infection prevalence or intensity associated with post-molt stages for larger crabs. In a recent study, no relationship was observed between infection prevalence and size in larger crabs, though the < 30 mm CW size class was not examined [21]. Hence, the relationship between molting, size, and infection by *H*. *perezi* remains to be elucidated.

This study assessed the hypothesis that juvenile crabs are more prone to infection, and if this was true, whether seasonal trends in prevalence and intensity were distinguishable relative to other size classes. The relationship between molt status and disease in blue crabs of >30 mm carapace width was also analyzed, using a radioimmunoassay that relies on ecdysone hormone levels to determine distinct molt stages [28]. Assessing the influence of these physiological factors provides critical insight into the susceptibility and timing of disease acquisition during the blue crab life cycle and how molting affects *H*. *perezi* proliferation in this crustacean host.

## Materials and methods

### Sample collection

Blue crabs were collected in conjunction with the Maryland Department of Natural Resources Finfish Investigation Surveys. These surveys are performed at 20 sites throughout the Maryland Coastal Bays (MCBs) from April through October (2014–2016) using an otter trawl as discussed previously in detail [14, 19]. Sampling typically began during the second week of the month and took place over the course of three non-consecutive days.

During sampling, live crabs were placed in bags labeled with the date and site and kept on ice for transport. All collected crabs were measured for carapace width (CW) and sexed upon arrival at the University of Maryland Eastern Shore Paul. S. Sarbanes Coastal Ecology Lab (Berlin, MD). Hemolymph (100 μL) was drawn from the arthrodial membrane of the 5^th^ walking leg using a 1 mL syringe equipped with a 27 gauge needle. Drawn hemolymph was immediately mixed in a 1:1 ratio of anticoagulant buffer (HEPES 10 mM, pH 7.4; NaCl 400 mM, KCl 10 mM, Glucose 100 mM, NaHCO_3_ 10 mM, EDTA 10 mM; [28]), and frozen at -50°C for future use.

Due to time constraints, occasionally larger crabs were frozen whole for processing at a later date. Crabs were also frozen if 100 uL of hemolymph could not be obtained (typically crabs < 40 mm CW). These crabs were frozen at -50°C and muscle tissue was dissected at a later date. All dissections and DNA purifications were performed at UMES main campus (Princess Anne, MD).

### DNA extraction

Once thawed on ice, 50 μL of hemolymph (1:1 dilution) was removed for DNA purifications. For smaller crabs, approximately 50 mg of muscle tissue was dissected from the large muscle in the body cavity associated with the 5^th^ pereiopod (swimmer fin). All purifications were performed using the Illustra Tissue and Cells Genomic Prep Kit (GE Healthcare), according to manufacturer’s protocols. Briefly, 50 μL of the 1:1 hemolymph and buffer mix was used, or 50 mg of muscle tissue, and resulting DNA from all purifications was suspended in 100 μL of the provided elution buffer. Tissue purifications were incubated at 56°C for 2 to 3 h, instead of the 1 h used for hemolymph samples. Each batch of purifications was run with a negative control (only the purification kit reagents were added) and a positive control, which received 50 μL of a plasmid containing the ribosomal DNA gene of *H*. *perezi*.

### Detection of *H*. *perezi* infections

A quantitative PCR assay was used to verify the presence or absence of *H*. *perezi* DNA in samples. The ITS2 primer set and protocol described in Hanif et al, 2013 [29] was used for all samples in this study. All qPCR assays were performed on a Bio-Rad CFX Connect Real-Time System using the Bio-Rad Universal Probe Sso Advanced Supermix. The negative control from each batch of purifications and a no template control were run in duplicate on each plate to ensure against contamination. The positive control from each batch of purifications was also run in duplicate on each plate to verify the presence of DNA in all samples.

Throughout this work, the presence or absence of the parasite is referred to as infection prevalence. Infection status is used to refer to the intensity of infection. As assayed and previously discussed [19], an estimated 300 copies of the ribosomal DNA gene per cell was used to calculate the number of *H*. *perezi* cells/mL hemolymph based on the gene copy numbers obtained from qPCR. Infection status was then determined from these estimates using the following categories as defined by Shields and Squyars, 2000 [30]: light infections (< 4.0 x 10^5^ parasites/mL), moderate infections (4.0 x 10^5^ to 2.0 x 10^6^ parasites/mL), and heavy infections (> 2.0 x 10^6^ parasites/mL).

### Size classes

In order to investigate variation in infection by size, crabs were grouped into four size classes; ≤ 30 mm CW (juvenile crabs), 31 to 60 mm CW (small crabs), 61 to 90 mm CW (medium crabs), > 90 mm CW (large crabs). These size classes are adapted from [14,27] which utilized six size classes, including 91 to 120 mm CW, 121 to 150 mm CW, and > 150 mm CW. These three large size classes were combined into one, as the larger size classes would have had relatively small sample sizes in this dataset.

### Ecdysteroid radioimmunoassay (Ecd-RIA)

When analyzing field-collected crabs, pre- and postmolt crabs are difficult to determine by visual inspection except for those that are very close to molt [31]. Thus, ecdysteroid levels in all available blue crab hemolymph samples were measured using RIA [28]. Each hemolymph sample (6 to 10 μL) consisted of 1:1 hemolymph and anticoagulant [32]. Samples were assayed in duplicate while standards were run in triplicate. Resulting data was analyzed using the AssayZap program (Biosoft). The data are presented as mean ± 1SE ng/mL hemolymph (n), where n is the number of animals. Molt stage classification were set as; 0 to 10 ng/mL hemolymph was considered postmolt, 10 to 30 ng/mL was considered intermolt, 30 to 70 ng/mL was considered early premolt, and > 70 ng/mL was considered premolt [28, 33, 34].

### Statistical analysis

Data from all three years was pooled in order to investigate broader trends. April and May were grouped into ‘spring’, June, July, and August were grouped into ‘summer’, and September and October were grouped into ‘fall’. Chi-square tests were used to investigate differences between categorical variables including the presence or absence of infection (referred to as infection prevalence), the intensity of infection (infection status), gender (as determined by apron shape), molt stage (as determined by ecdysone concentration), size class, month, and year.

The chi-square goodness of fit test was used to determine if differences existed between infection prevalence in the molt subsample and the total dataset. For this test, the proportions of infected and uninfected crabs in the total dataset were used as the expected values and the proportions of infected and uninfected crabs in the molt subsample were used as the observed values.

Student’s t-tests were used to explore the relationship between ecdysone and infection for all pooled data and by month. The assumption of normality was not met for ecdysone concentrations, as determined by Shapiro tests on the entire dataset and individual months. However, ecdysone concentration between groups (infected vs. uninfected crabs) was skewed in the same direction indicating the t-test was likely not influenced by this deviation from normality [35]. In addition, the assumption of equal variance between groups was met as determined by Levene’s tests.

All tests were considered significant at p < 0.05. Statistical tests were performed in R (version 3.3.3) using R studio (version 1.0.136) [36, 37].

## Results

### Blue crabs sampled

During the course of this three year study, a total of 1037 blue crabs were assayed for the presence of parasite DNA from both tissue and hemolymph samples depending on size and availability of 100 μL of hemolymph. These crabs ranged in size from 11 to 157 mm CW, with an average size of 56.4 mm CW. Of the crabs sampled, 609 were male (58.7%) and 428 were female (41.3%).

### Infection prevalence

Using qPCR, 427 (41.2%) crabs tested positive for parasite DNA, which indicated positive infection status [38]. No significant difference was seen in infection prevalence by gender (p = 0.46). Overall infection prevalence varied significantly by year (chi-square analysis, X^2^ = 61.0, df = 2, p < 5.6x 10^−14^), with 2015 and 2016 being relatively high infection years and 2014 being a relatively low infection year. In 2014, 19.3% of all crabs tested were infected (44 of 232), as opposed to 48% (144 of 297 crabs) in 2015 and 47% (239 of 508 crabs) in 2016.

**Fig 1:**
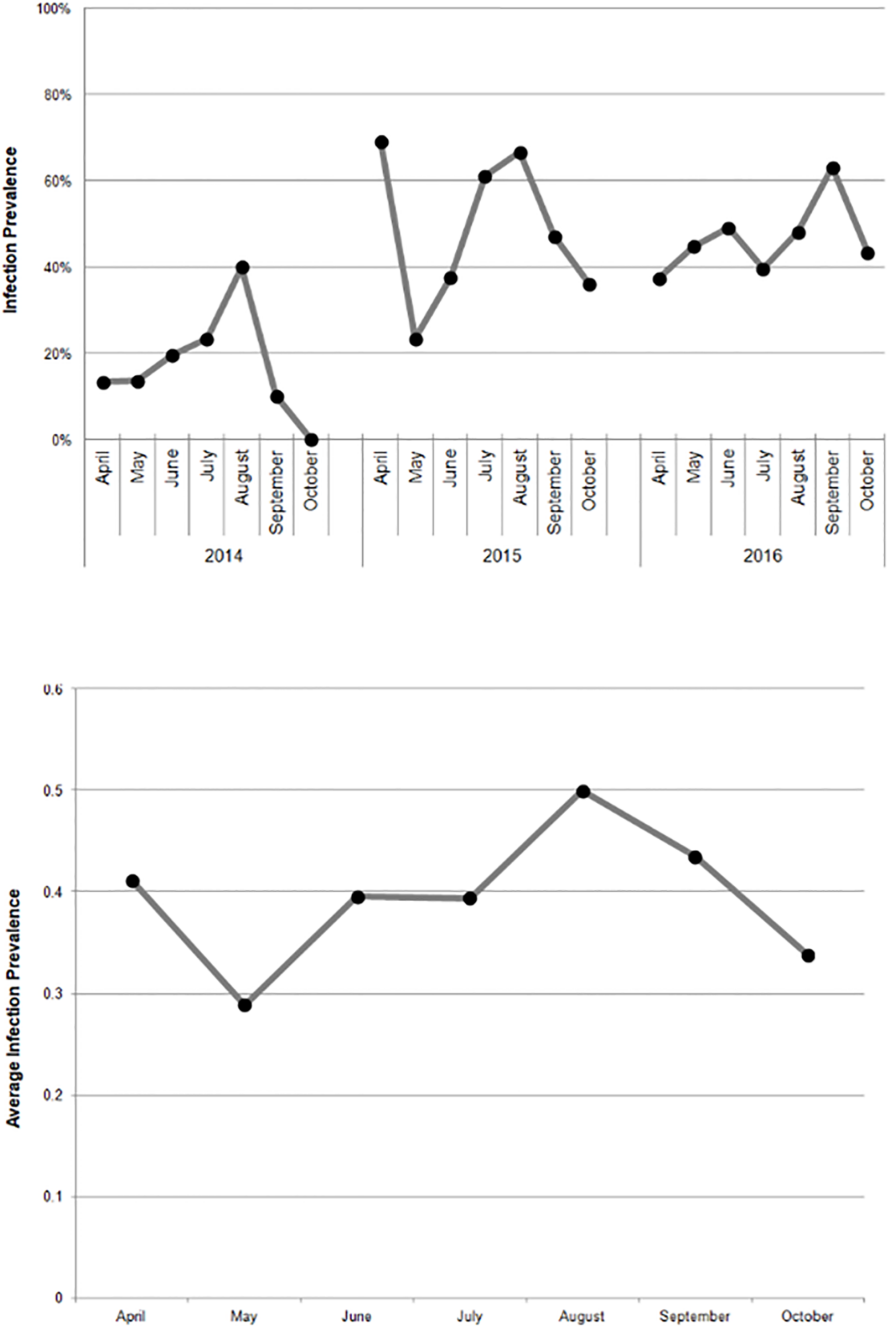
Monthly infection prevalence 2014–2016. The percent of infected crabs (infection prevalence) is graphed for each month where samples were collected from the Maryland coastal bays. Figure modified from [19]. (A) Each individual year is shown in chronological order. (B) Pooled dataset.

Within each year, disease varied over time with infection prevalence and intensity typically peaking in summer. In all three years, infection prevalence was significantly different by month (X^2^ = 18.7, df = 6, p < 0.005; X^2^ = 27.5, df = 6, p < 0.0002; X^2^ = 13.1, df = 6, p < 0.05, respectively). In addition to the summer peak, a variable spring peak occurred in 2015 and 2016 (Fig 1A), though the timing and intensity of this peak differed between years. When monthly infection prevalence was pooled across all three years, August was shown to be the peak month of infection (Fig 1B).

### Infection and size

One of the primary goals of this work was to investigate whether a particular size class was more or less susceptible to harboring disease. Significant differences were observed in 2015 for both infection prevalence and infection status by size class (X^2^ = 23.5, df = 3, p < 3.2 x 10–5; X^2^ = 27.9, df = 9, p < 0.001, respectively). However, no difference in infection prevalence by size class was seen in this dataset for 2014 or 2016 (p = 0.17; p = 0.46, respectively), nor was there a difference in infection status by size class (p = 0.66; p = 0.22, respectively). Data for all three years was thus pooled, in order to better represent the system in the absence of yearly fluctuations. Infection intensity by size class was significantly different in this pooled dataset (X^2^ = 30.9, df = 9, p < 0.0004; Table 1). The infection prevalence for all crabs collected was 41.2%. Small crabs (31–60 mm CW) were the least likely to be infected, with 34.4% testing positive for *H*. *perezi* (143 out of 416). Interestingly, the next size class of medium crabs (61–90 mm) was most likely to harbor infection (100/202, or 47.2% prevalence), followed closely by juvenile crabs (≤30 mm) at 46.7% prevalence (105/225). Very few crabs <60 mm CW harbored *H*. *perezi* with high intensity. By contrast, medium and large (> 60 mm CW) crabs were most likely to have advanced infections, with 9.0% (19/212) of medium crabs and 9.8% (18/184) of large crabs having moderate and heavy intensity infections.

**Table 1:** Pooled infection status (intensity) by size class.

	≤ 30	31 to 60	61 to 90	> 90	Total
No	120 (53.3%)	273 (65.6%)	112 (52.8%)	105 (57.1%)	610
Light	95 (42.2%)	133 (32.0%)	81 (38.2%)	61 (33.1%)	370
Moderate	6 (2.7%)	7 (1.7%)	12 (5.7%)	9 (4.9%)	34
Heavy	4 (1.8%)	3 (0.7%)	7 (3.3%)	9 (4.9%)	23
**Total**	225	416	212	184	1037

The number of crabs in each infection status by size class is displayed. The number in parentheses represents the proportion of crabs with that infection status in a given size class.

### Infection, size, and season

A second goal of this work was to determine if infection prevalence and infection intensity would vary for a given size class over time. Sub-dividing the dataset by season permitted an exploration into changes throughout the year, while still maintaining a sufficient sample size for statistical analysis. The seasonal breakdown used for this study was based on monthly average water temperatures in the MCBs and grouped the cooler months early in the year as spring (April and May), the warmest months as summer (June, July, and August), and the cooler months later in the year as fall (September and October).

This more detailed analysis showed that infection did vary by size class and season (Table 2). This data shows crabs shifting from smaller size classes early in the year to larger size classes later in the year, which follows observed growth patterns in the Chesapeake Bay [39]. Juvenile (≤ 30 mm CW) crabs were not well represented in the fall, but had higher infection prevalence than the overall infection prevalence for that season (62.5% of juvenile crabs [10/16] were infected vs. 40.7% [92/226] of all crabs). For all three seasons, small crabs tended to have lower infection prevalence than other groups, and the majority of those infections were classified as light. Medium crabs were scarce in spring months and had lower infection prevalence than overall spring infection prevalence (16.7% [2/12] of medium crabs were infected vs. 36.3% [82/226] of all crabs). However, infection prevalence in this category increased from 16.7% to 54.5% (61/112) in the summer, the largest rise in any size category for any season. Moderate and heavy infections were primarily found in crabs > 60 mm in size and occurred throughout the year.

**Table 2:** Infection status (intensity) by size class and season.

**Spring**	≤ **30**	**31 to 60**	**61 to 90**	**> 90**	**Total**
No	52 (57.1%)	60 (75.9%)	10 (83.3%)	22 (50.0%)	144
Light	36 (39.6%)	17 (21.5%)	1 (8.3%)	20 (45.5%)	74
Moderate	2 (2.2%)	1 (1.3%)	1 (8.3%)	0	4
Heavy	1 (1.1%)	1 (1.3%)	0	2 (4.5%)	4
**Total**	91 (40.2%)	79 (35.0%)	12 (5.3%)	44 (19.5%)	226
**Summer**	≤ **30**	**31 to 60**	**61 to 90**	**> 90**	**Total**
No	62 (52.5%)	173 (63.8%)	51 (45.5%)	46 (54.8%)	332
Light	50 (42.5%)	92 (34.0%)	47(42.0%)	28 (33.3%)	217
Moderate	3 (2.5%)	5 (1.8%)	9 (8.0%)	4 (4.8%)	21
Heavy	3 (2.5%)	1 (0.4%)	5 (4.5%)	6 (7.1%)	15
**Total**	118 (20.2%)	271 (46.3%)	112 (19.1%)	84 (14.4%)	585
**Fall**	≤ **30**	**31 to 60**	**61 to 90**	**> 90**	**Total**
No	6 (37.5%)	40 (60.6%)	51 (58.0%)	37 (66.1%)	134
Light	9 (56.2%)	24 (36.4%)	33 (37.5%)	13 (23.2%)	79
Moderate	1 (6.3%)	1 (1.5%)	2 (2.3%)	5 (8.9%)	9
Heavy	0	1 (1.5%)	2 (2.3%)	1 (1.8%)	4
**Total**	16 (7.1%)	66 (29.2%)	88 (38.9%)	56 (24.8%)	226

The number of crabs in each infection status, by size class, is displayed for spring (April and May), summer (June, July, and August), and fall (September and October). The number in parentheses represents the proportion of crabs with that infection status in a given size class. In the rows showing the total number of crabs in each size class by season, the percentage in parentheses represents that total proportion of the population in that size class and season.

### Molt stage

**Fig 2:**
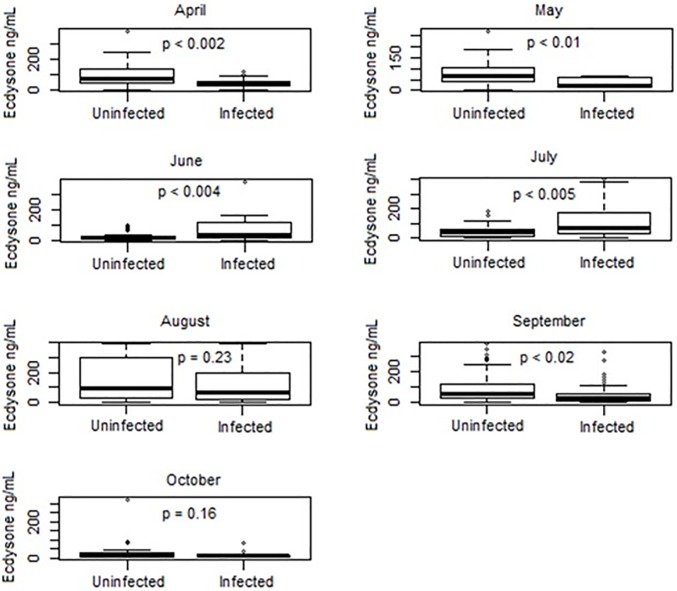
Ecdysone concentration by month for infected and uninfected crabs. Monthly boxplots of pooled ecdysone concentrations for uninfected and infected crabs are shown. The p-values listed corresponds to the t-test of ecdysone concentration between infected and uninfected crabs for each month.

In order to explore the relationship between molt stage, size, and disease, a subsample of the total dataset was utilized for the Ecd-RIA. Because only hemolymph samples were available for this method, 502 crabs were analyzed, ranging in size from 30 mm CW to 157 mm CW. From this subsample, a total of 227 crabs (45.2%) were positive by qPCR analysis. Although slightly higher, this was not significantly different from the overall prevalence (41.2%) in the complete dataset (p = 0.13). Additionally, no significant differences were observed between infection prevalence in individual size classes between the molt subsample and the total dataset (p = 0.23, p = 0.55, and p = 0.30 for the three size classes present in the molt subsample; 30 to 60 mm CW, 60 to 90 mm CW, and > 90 mm CW, respectively). A t-test showed that there was no difference in ecdysone concentration between infected and uninfected crabs over the entire sampling season of a pooled dataset (t = 0.454, df = 477, p = 0.65). However, when crabs were analyzed by month, significant differences became apparent (Fig 2). In April, May, and September, uninfected crabs had significantly higher concentrations of ecdysone (t = 3.33, df = 37.2, p < 0.002; t = 2.82, df = 24.3, p < 0.01; t = 2.39, df = 118, p < 0.02, respectively). Interestingly, June and July showed an inverse relationship, with infected crabs having significantly higher concentrations of ecdysone (t = -3.08, df = 40.8, p < 0.004; t = -2.96, df = 43.2, p < 0.005, respectively). August and October showed no significant differences between infected and uninfected crabs (p = 0.23; p = 0.16, respectively).

### Disease and ecdysis

Crabs were further subdivided into specific molt stages according to ecdysone levels, as previously defined [28]. During individual years of the three year study, infection prevalence was not significantly different by molt stage (p = 0.77; p = 0.49; p = 0.28, respectively), nor was there any difference in the pooled dataset (p = 0.82). Infection intensity by molt stage was significantly different in 2016 (X^2^ = 24.1, df = 9, p < 0.0041), but this was not the case for 2014 or 2015 (p = 0.76; p = 0.29, respectively). When the data for all three years was pooled, a relationship between molt stage and infection status was observed (X^2^ = 19.4, df = 9, p < 0.03). Higher proportions of crabs in early premolt and premolt were uninfected, with higher proportions of postmolt crabs harboring moderate and heavy infections (Table 3). Further analysis by season (Table 4) demonstrated that of the 29 crabs detected in the premolt stage in the spring, only 13.8% (4/29) were infected, as compared to 48.1% (26/54) of crabs in other stages. By contrast, premolt stages in the summer had 58.3% disease prevalence (63/108), with 51.1% prevalence (71/139) in other stages. By fall, infection prevalence had reverted to the same pattern observed in the spring with 26.7% (12/45) of premolt crabs being infected, as opposed to 40.2% (51/127 crabs) of crabs in other stages. We conclude that uninfected crabs are more able to reach ecdysis, with an apparent delay in molt progression observed in infected crabs in the spring. For crabs that are infected and do enter ecdysis, a phenomena that was most pronounced in the summer, proliferation of the parasite appears to be enhanced.

**Table 3:** Infection status by molt stage.

	Postmolt	Intermolt	Early Pre	Premolt	Total
No	37 (52.1%)	63 (52.1%)	72 (56.3%)	103 (56.6%)	275
Light	20 (28.2%)	51 (42.1%)	44 (34.4%)	69 (37.9%)	184
Moderate	8 (11.3%)	2 (1.7%)	5 (3.9%)	7 (3.8%)	22
Heavy	6 (8.4%)	5 (4.1%)	7 (5.4%)	3 (1.6%)	21
**Total**	71	121	128	182	502

The number of crabs in each infection status by molt stage is displayed. The number in parentheses represents the proportion of crabs with that infection status in a given molt stage.

**Table 4:** Molt stage by season.

Pooled	Spring	Summer	Fall
**Post**	9 (44.4%)	27 (48.1%)	35 (48.6%)
**Inter**	11 (54.5%)	66 (50.0%)	44 (43.2%)
**Early**	34 (47.1%)	46 (54.3%)	48 (31.3%)
**Premolt**	29 (13.8%)	108 (58.3%)	45 (26.7%)
**Total sampled**	83	247	172
**Total infected**	30 (36.1%)	134 (54.3%)	63 (36.6%)

The number of crabs in each molt stage is displayed for spring, summer, and fall. The number in parentheses represents the percent of crabs infected in each category. Note that these percentages are different from the other tables presented.

## Discussion

### Juvenile crab infection and season

Previous work in the MCBs noted higher infection prevalence of *H*. *perezi* in juvenile crabs and this was hypothesized to be linked to the increased molt frequency within the size class [14, 20]. Although it was not possible to assess the molt frequency of these animals using Ecd-RIA, juvenile crabs did have a relatively high infection prevalence of 46.7% (Table 1) and displayed their highest infection prevalence in fall at 62.5% (Table 2). This is intriguing in light of recent work which showed that naïve crabs ≤30 mm, deployed as sentinels, can rapidly acquire infections in September at a prevalence upwards of 85% [40]. A previous study also showed that juvenile crabs (collected between summer of 1992 and fall of 1993) had higher infection prevalence in the fall as compared to spring, where levels were non-detectable by microscopy [27]. Future work should thus be directed at determining if juveniles are in fact more susceptible to infection in the fall, as average water temperatures are higher than in spring and may be a causative factor [19]. Juvenile crabs present in the fall likely originate from a late May/early June spawning event within the same year, whereas spring juveniles derive from spawning that occurs either in early fall or from slower-growing recruits from the summer spawning cycle of the previous year [39]. This raises the possibility that disease may be related to reproductive cycles in blue crabs; specifically, the timing of when crabs spawn may be important in explaining the yearly variation in prevalence (Fig 1).

### Size class and disease progression

One assumption in interpreting this dataset is that new infections will initially present as low intensity and progress into more advanced stages, as has been previously shown [14, 30]. In the work presented here, crabs between 30 and 90 mm CW showed increased infection prevalence between spring and summer, with most of these animals acquiring infections categorized as light (Table 2). Thus, the most straightforward interpretation is that animals in this size class (which also represent the bulk of the blue crab population in this time period) are most susceptible to acquiring disease in the summer, likely related to the increased abundance of presumptive dinospores during summer months [19]. Summer is when crabs in the >60 mm size class had the highest degree of moderate and heavy infections, supporting the observation that disease progression can be rapid under the right conditions [30]. Our data also provides statistical support for concluding that, although all size classes are capable of harboring each intensity level of infection, parasite proliferation is enhanced in larger animals, contrary to what had been previously reported [14].

### Molting and disease progression

The blue crab spring molt cycle has been described as “synchronous,” due to a reset of the cycle that occurs in winter, when temperatures drop below a metabolic temperature minimum (T_min_) of 8.9 °C [41]. As temperatures climb above T_min_, all crabs are theoretically at the same point (the “synchrony point”) in the molt cycle [31]. Crabs within the same instar (the number of molts since hatching), theoretically molt on the same time scale, with the acknowledged caveat that animals in the field are subject to varying ecological conditions such as temperature fluctuations, prey availability, health, etc. Thus, while the concept of a synchronous molt is an oversimplification of an observed phenomenon, it remains likely that spring is when the clearest relationship between molting and infection should be observed. Accordingly, from 2014 to 2016 in the months of April and May, uninfected crabs had significantly higher concentrations of ecdysone as compared to infected crabs (Fig 2). Interestingly, infected crabs appear to have a delayed molt in the spring, while in the summer there is no apparent inhibition in this process (Table 4).

In an experimental infection study, increased parasite density was observed in infected hosts when water temperature was raised from 12° to 16°, although the effect of even higher temperatures was not assessed [29]. *In vitro* culturing studies have shown that *H*. *perezi* will only develop into sporoblasts (the precursor to the generation of dinospores) when cells are grown at 23° and not at 15° [9]. Collectively this previous work, along with the data presented here, supports the idea that summer is the optimal growth period for *H*. *perezi* in the MCBs. This would also coincide with a period when the host’s metabolism is optimal. That dinospore occurrence and density peaks in these months [19] hints at the process of a spring molt delay as an evolutionary adaption by the parasite, likely related to optimizing its temperature range for proliferation and dispersal.

Smith and Rowley, 2015 [42] reported that infected *C*. *pagurus* molted less frequently than uninfected crabs of the same size, suggesting that the phenomena observed in this study may not be unique to blue crab hosts. Examples of manipulation of host molting have been observed in other invertebrate systems, such as in the baculovirus group that infects moth larvae [43]. In *Autographa californica* Nuclear Polyhedrosis Virus (AcPNV), expression of an ecdysteroid UDP-glucosyltransferase has been shown to inhibit molting by inactivating ecdysone, leading to an increase in time to death and host weight [44, 45]. In this system, it is thought that this inhibition allows the host to continue feeding, which then supports increased viral load and subsequent transmission upon host death [45].

The mechanism by which *H*. *perezi* reduces ecdysteroid levels, and whether they utilize this steroid for metabolic purposes, should be the focus of future studies. The related oyster parasite, *Perkinsus marinus*, is unable to synthesize cholesterol, and must therefore obtain it from its host for membrane synthesis [46]. It may be possible that *Hematodinium* spp. competes with the host for ecdysteroid by upregulating competitive ecdysteroid-specific receptors, in order to recycle this metabolite into its cholesterol synthesis pathways. Currently, a fully annotated *Hematodinium* spp. genome and transcriptome are not available, but the publication of such would suggest whether such a system is possible, in addition to elucidating other potential pathways involved in lowering ecdysteroid levels in infected hosts.

Regardless of when molting eventually occurs, there was a statistically significant enhancement in parasite load in postmolt blue crabs (Table 3). This is consistent with what has been observed in *Hematodinium* spp. infection in *Chionoecetes* spp. [11, 24] and Norway lobsters [47]. Ecdysis is a metabolically demanding event. Under normal, presumably non-infected conditions, crabs are known to release crustacean hyperglycemic hormone (CHH) in order to increase the available energy (glucose) that is necessary for the ecdysis process [48]. During post molt, when crabs are not feeding, transcriptomic analysis has demonstrated the up-regulation of gluconeogenic genes such as phosphoenol pyruvate carboxykinase, resulting in greater production of glucose [49]. The depletion of host metabolic reserves is a known phenomenon in *Hematodinium* spp. infections and is thought to influence mortality [47, 50, 51]. This leads to the hypothesis that *Hematodinium* spp. utilize the post molt period as a particularly beneficial window for enhanced replication. Future studies should therefore be directed at measuring a battery of metabolic properties in infected and non-infected hosts, through all molt stages, with a particular emphasis on glucose-related pathways. Glucose is also an important precursor to chitin synthesis, and the cuticle protein *cbm* (thought to be involved in shell hardening) is upregulated in post molt animals [49]. The assessment of post-molt shell hardness, and time to completion of shell hardening, may be particularly important analyses in experimental *Hematodinium* spp. infections, as this disease may make animals more vulnerable to predation in their natural setting.

## Conclusions

The work presented here sought to resolve the question of what role, if any, do size class and molt status have in the *H*. *perezi* disease cycle. Ultimately, size does appear to be an important factor in disease, although this varies between seasons. Juvenile crabs appear to be more susceptible to disease in the fall, medium sized crabs are more susceptible to initial infection in the summer, and proliferation of the parasite is favored in crabs >60 mm.

Molt stage and infection by *H*. *perezi* also appear to be related. However, the data presented here suggests that the molt process may not be a primary pathway for the parasite to gain entry into the host as previously thought. Rather, it appears that the parasite may delay the molt cycle in the spring in order to synchronize host and parasite metabolism. This may then allow for disease progression into more advanced (moderate and heavy intensity) stages.
